# Detection, Diagnosis, and Monitoring of Early Caries: The Future of Individualized Dental Care

**DOI:** 10.3390/diagnostics13243649

**Published:** 2023-12-12

**Authors:** Marwa Abdelaziz

**Affiliations:** Division of Cariology and Endodontology, Department of Preventive Dental Medicine and Primary Care, University Clinics of Dental Medicine, University of Geneva, Rue Michel-Servet 1, 1211 Geneva, Switzerland; marwa.abdel@unige.ch

**Keywords:** caries, diagnosis, detection, near infrared transillumination, fluorescence

## Abstract

Dental caries remains a significant global health issue. It was highlighted by the World Health Organization’s 2022 reports that despite the efforts and scientific advancements in caries detection and management, the situation has only marginally improved over the past three decades. The persistence of this problem may be linked to outdated concepts developed almost a century ago but are still guiding dentists’ approach to caries management today. There is a need to reconsider professional strategies for preventing and managing the disease. Contemporary dentistry could benefit from embracing new concepts and technologies for caries detection and management. Dentists should explore, among others, alternative methods for caries detection such as optical-based caries detection. These tools have been established for over a decade and they align with current disease understanding and international recommendations, emphasizing early detection and minimally invasive management. This narrative review presents the current state of knowledge and recent trends in caries detection, diagnosis, monitoring, and management, offering insights into future perspectives for clinical applications and research topics.

## 1. Introduction

The 2017 Global Burden of Disease study, as reported by The Lancet, revealed that out of 328 diseases examined, dental caries in permanent teeth had the highest prevalence, and its incidence ranked second. Shockingly, over one-third of the world’s population is living with untreated dental caries. When it comes to deciduous teeth, untreated caries stands as the most prevalent chronic childhood ailment, impacting a staggering 514 million children globally [1].

This clearly suggests that our efforts need to shift focus from how we treat the symptoms of the disease (i.e., tooth cavities, discoloration, pain, etc.) to how to prevent it and manage the early symptoms (i.e., early lesions, biofilm dysbiosis, etc.).

Establishing dentistry as a surgical profession early in the 20th century seems to have determined a mainly operative approach toward managing dental caries. This approach was initially based on the necessity to treat extensive caries and associated pain or infection with the limited means available during that period [2]. Currently, the presentation of the disease has changed with the wide adoption of oral hygiene and the use of fluoridated toothpaste. Nowadays we mostly deal with slow-progressing, early, non-cavitated caries [3,4]. However, the restorative-focused clinical practice today does not reflect the current knowledge regarding the effectiveness of early prevention or non-invasive management strategies [5,6].

The key problem seems to be that operative care has remained the central focus for the caries control strategy communicated in dental schools and practiced by most dentists [7].

The understanding of dental caries has evolved over time. Initially seen as a simple and irreversible process, treatment focused on removing affected tissue, as per G.V. Black’s “extension for prevention” concept [8]. In 1935, enamel “white spots” were discovered, initially thought to be photographic artifacts [9]. Research in the 1940s and 1950s contributed to understanding early enamel lesions [10,11]. By the 1970s, dental caries was defined as a multifactorial infectious disease, with the disease process linked to bacterial biofilm on the tooth surface. This model considered demineralization as a reversible sign of active disease, leading to an established understanding by the late 1980s [12].

According to Fejerskov et al., 2015 “The term dental caries is used to describe the results (the signs and symptoms) of a localized chemical dissolution of the tooth surface caused by metabolic events taking place in the biofilm (dental plaque) covering the affected area” [13].

Recently, dental caries was classified by the World Health Organization (WHO) as “a plaque (biofilm)-mediated, non-communicable disease (NCD), with a complex network of biological, genetic, behavioral, socioeconomic, and lifestyle-related risk factors in common with other NCDs, for example, obesity and diabetes” [14]. This implies that we should not anticipate a single risk factor, like bacterial load, sugar consumption, or salivary secretion rate, to be autonomously effective in managing the disease or forecasting future caries occurrences.

New approaches to managing dental caries have emerged over the past few decades to adapt to the evolving understanding of the disease, sparking intense debates and discussions regarding how to address this issue, both in terms of prevention and treatment. 

One major concept proposed for caries management is grounded in the understanding of caries as a non-communicable disease closely linked to individual behaviors and lifestyles [15].

Traditional preventive measures, such as improving oral hygiene, using fluoridated toothpaste, applying topical fluoride, and modifying the diet of individuals showing early signs of caries, have conventionally been labeled as “prevention.” However, this term may not accurately describe the process, as it does not entirely prevent caries. Instead, it primarily aims to slow down or delay the progression of non-cavitated lesions into cavitated ones [13,16].

Regrettably, the term “prevention” has often been juxtaposed with “treatment,” where “treatment” refers to operative interventions like drilling and restoration. Many patients, dentists, and policymakers tend to favor operative interventions as the primary means to manage caries, without considering that once a tooth is drilled and restored, it initiates a restorative cycle that increases the likelihood of tooth loss with age [17]. 

The conventional approach encouraged dentists and patients to achieve a fully restored dentition with no visible signs of the disease, setting it as the gold standard. This has made it challenging to accept that not all lesions are active or present a health risk to the patient. Recognizing that arrested lesions can be viewed as “scars” has the potential to alter how dentists conduct clinical examinations and approach caries management.

Today it is clear that dentists’ understanding and practice of caries detection, assessment, and management need to adopt new concepts and technologies available. 

This review aims to present and discuss the evidence surrounding contemporary concepts in caries detection and management, with a specific focus on modern optical-based methods such as near-infrared transillumination and laser fluorescence imaging. The review will explore how these technologies, when integrated with non-invasive and minimally invasive treatments like sealing and infiltration of early lesions, align with modern caries management principles and international guidelines. The emphasis is on fostering a patient-oriented, risk-based, preventive, and non-invasive approach to manage the disease and not only the lesion.

The review will provide a perspective on a comprehensive caries management approach that integrates evidence from caries detection, activity assessment, and patient risk assessment. Ultimately, it encourages readers to reflect on the current landscape and highlights the imperative for future research to support personalized dental care.

To elaborate this review, studies from the past decade covering this subject were retrieved from electronic databases such as PubMed, Scopus, and Science Direct.

## 2. Caries Terminology, Severity, and Activity Assessment

Understanding and using clear terminology of dental caries for teaching, communication, and reporting research is essential [18]. 

Different classifications exist, and they are mostly based on location, depth, and clinical aspects [3,19,20]. Recent therapeutically driven classifications of primary caries, consider that the focus point must be the determination of the lesion activity and the enamel surface integrity [21].

Upon clinical examination, with the goal of establishing a care plan for the patient, it must be kept in mind that this assessment should be weighed on many levels: patient’s level, oral level, tooth level, and surface level. 

### 2.1. Caries Lesion Severity Assessment

The assessment of caries lesion severity offers a way to categorize the progression of net mineral loss, starting with small lesions and advancing to greater levels of tooth damage that can extend through the enamel and dentin and even involve the dental pulp. This evaluation can be conducted through various classification methods and systems. For instance, it can be classified clinically into stages such as non-cavitated, micro-cavitated, and cavitated lesions [22,23], clinical and radiographic staging into initial, moderate, and extensive lesions [20], and clinical staging from non-cavitated lesions to pulpal sepsis [24]. 

#### 2.1.1. Non-Cavitated Lesions

They are also known as pre-cavitated lesions, early lesions, incipient lesions, superficial caries, or typically the “white spot” lesions. The white appearance is mainly due to the enamel surface porosity and changes in its mineral content. The dissolution may even reach the dentin and provoke a reaction in the dentin long before cavity formation [13]. 

It is crucial to avoid using a sharp probe when conducting a clinical exam. Applying pressure with the sharp tip may cause irreversible damage to the demineralized enamel surface and render a reversible lesion into a cavitated lesion [25,26]. Using a dull-ended probe with gentle pressure is recommended.

Non-cavitated lesions may be active or arrested, the management is different for each type. The distinction is mostly based on the clinicians’ judgment of the lesions’ clinical features, combining it with an assessment of the oral health status of the patient [13,27] 

Non-cavitated lesions may be arrested by means of remineralization using fluoride and calcium-based products or—if the non-invasive management approach is not effective- by isolating the lesion from the biofilm using resin to seal or infiltrate the lesion. Sealing and infiltration can provide protection against further demineralization by creating a diffusion barrier against the acids causing the demineralization [3,28].

#### 2.1.2. Cavitated Lesions

When tissue destruction continues, the enamel loses its mechanical resistance and a surface breakdown occurs, causing a micro-cavity, localized surface defect in enamel only without undermined enamel. This enamel discontinuity will progress by exposing dentin and establishing a lesion that is referred to as a “cavitated lesion” [13].

A cavitated lesion with exposed dentin is more difficult to clean as it will retain an established biofilm on its surface, making its progression inevitable. Such a lesion is considered irreversible and requires a restorative treatment as the treatment of choice, unless in some situations the biofilm can be managed appropriately, such as vestibular accessible lesions [29,30,31]. 

While cavitations on occlusal and vestibular lesions are easy to asses by clinical examination, cavitation of proximal lesions has been relying on lesion depth on the bitewing X-rays. Even though earlier studies showed that 100% of proximal lesions reaching the inner half of dentin on the bitewing radiographs are cavitated [32,33], further studies established that only one of three lesions in the first third of dentin is cavitated [31]. 

Cavitated lesions are classically treated by restorative fillings after the excavation of the carious tissue. In some situations, the sealing could arrest cavitated lesions, as the seal deprives the bacteria in the lesion of dietary carbohydrates [34,35]. This is explained by the fact that the sealed bacteria are no longer cariogenic because their metabolic activity is reduced the lesion is arrested [36,37].

Moderate lesions with micro cavitations in the enamel may be sealed; however, guidelines state that for cavitated lesions (exposed dentin), a full excavation is recommended to ensure the longevity of the restoration. Hard clean enamel and dentin periphery are maintained while leaving re-mineralizable dentin in the center for deeper lesions [38].

### 2.2. Caries Lesion Activity Assessment 

To establish personalized, minimally invasive oral care, it is crucial to categorize lesions as either “arrested” or “active” and to continually monitor their status. There are only a few validated systems for assessing lesion activity, including the International Caries Detection and Assessment System (ICDAS) [39] and the Nyvad criteria [40], which rely on the lesion’s clinical characteristics.

Caries activity reflects the mineral balance over time, encompassing net mineral loss, net mineral gain, or stability. It serves as an indicator of caries initiation or progression, while caries inactivity suggests that the caries process has come to a halt (regression) [41]. The assessment of caries lesion activity aims to distinguish between active and inactive lesions, facilitating optimal care planning by dentists with an emphasis on halting active lesions. Even inactive lesions and healthy tooth surfaces should receive routine care and appropriate monitoring [18].

#### 2.2.1. Active Lesions

Evaluating a lesion’s activity will take into consideration multiple factors such as color, location, and texture, as well as some clinical aspects not directly related to the lesion itself. 

**Figure 1:**
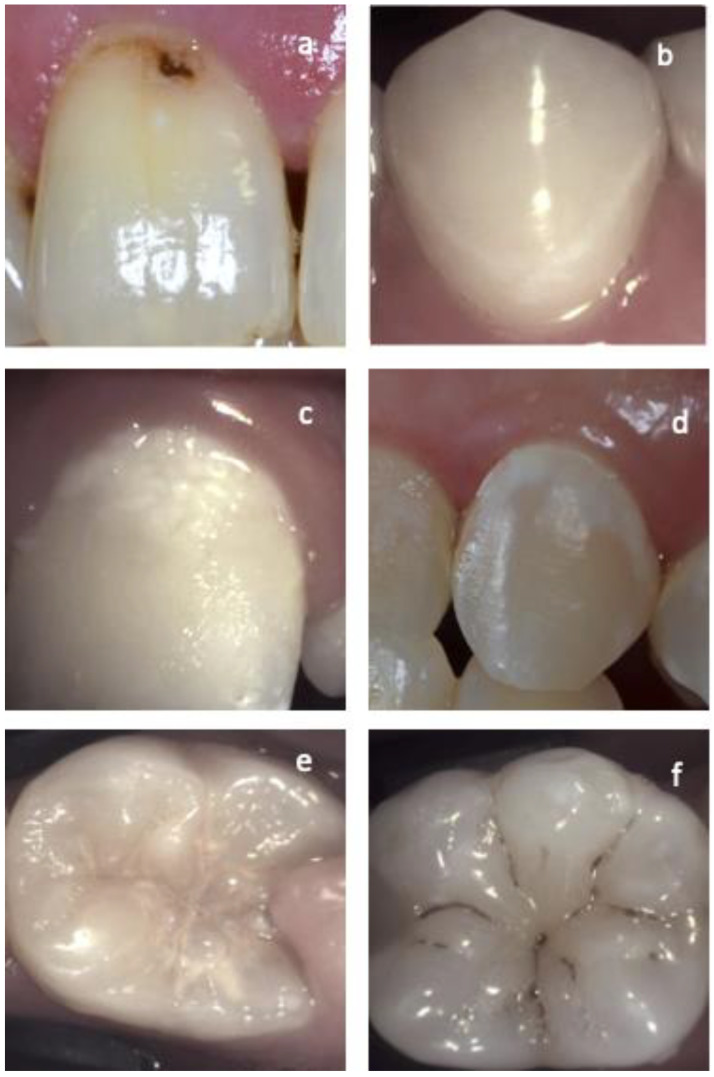
Different examples of active and arrested lesions on vestibular and occlusal surfaces. (**a**) Arrested cavitated enamel lesion. Notice the dark color, lack of plaque on the surface, and the band of healthy enamel adjacent to the gingiva line. (**b**) Arrested non-cavitated enamel lesion on the vestibular surface. Lesion in-activity is confirmed by the clean shiny smooth surface and the healthy enamel separating the lesion from the gingiva in contrast with images (**c**,**d**) where active non-cavitated lesions can be observed. The difference between lesion activity on occlusal surfaces is demonstrated in the images. (**e**) Active occlusal lesion and (**f**) arrested occlusal lesion.

For an active pre-cavitated enamel lesion, the surface of the enamel is whitish/yellowish opaque with a loss of luster (Figure 1d,e), and it feels rough when the tip of the probe is moved gently across the surface. Active lesions are generally covered with plaque (Figure 1c) [21,42].

Smooth surface caries lesions are typically located close to the gingival margin (Figure 1d), and just like for proximal lesions, the presence of plaque and the bleeding on probing of the adjacent gingival area is a strong indicator of lesion activity [21,42].

For occlusal caries, intact fissure morphology is observed with the lesion extending along the walls of the fissures (Figure 1e) [21,42].

Depending on lesion activity, the microbiological profile seems to shift. Yet, studies show that there are very few bacteria observed in the body of non-cavitated lesions and they do not show an established biofilm [29,30,31,43]. 

#### 2.2.2. Arrested Lesions

When the enamel lesion is arrested, the surface of the enamel is whitish, brownish, or black (Figure 1a,b,f). Enamel may be shiny and feels hard and smooth when the tip of the probe is moved gently across the surface. A smooth surface lesion shows a thin line of healthy enamel separating the lesion from the gingiva. A clean surface with no plaque accumulation or adjacent gingival bleeding is another indicator of lesion inactivity [21,42]. Arrested cavitated dentin lesions are mostly dark and the dentin is shiny and hard on gentle probing [40].

It must be kept in mind that caries development is a dynamic process. Just like active caries can be arrested, the process could happen the other way around, especially when considering that the main causing factor that influences the microbiological balance is the patient’s cooperation (diet, maintaining oral hygiene, plaque control, using remineralizing agents). Without protection or proper management, active non-cavitated lesions progress into cavitated lesions [44,45].

## 3. The Paradigm Shift in the Understanding and Management of Dental Caries

Understanding caries has extensively evolved in the past century. In the early 1900s, the disease was understood and managed as an infectious disease [46]. This understanding led to the aggressive management to remove all infected tissue (extension for prevention). 

Later, around the 1950s, the relationship between biofilm, sugar, and caries development was better established [47]. Around the same time, the important realization that bacteria and sugar are required together to initiate caries led to the development of the preventive approach including the introduction of fluoride [48,49].

The focus on the biofilm and its complexity started in the late 1980s, leading to the actual understanding of the important concept of dysbiosis and the understanding of caries as a non-communicable disease today [15,50]. The understanding of this model is one of the main points that caused the paradigm shift in cariology research and practice in the last decade [3,28]. The introduction of sealing caries to create a barrier between the bacteria and the oral environment was a revolutionary approach after years of practice based on the extension for prevention model.

The recent changes in the understanding of the disease added to the reduction in sever caries prevalence [51], slow carious lesion progression [52], the limitations of visual, tactile, and radiographic examination, combined with the increased emphasis on using less ionizing radiation and the focus on early caries detection and management [7,28,53], have pushed researchers to investigate the potential of alternative less invasive management approach.

To provide more preventive and non-invasive dental care, it is essential to detect carious lesions as early as possible, preferably when still within the enamel to increase the success chance of non-invasive and micro-invasive management. To achieve this goal, alternative caries detection methods with higher sensitivity for initial lesion detection and monitoring must be considered [31,54]. Detection methods such as optical-based caries detection methods to diagnose early lesions were developed and intensively investigated over the past 20 years [55,56,57].

The management of dental caries has also shifted over the past decade. Instead of basing the treatment decision entirely on radiographic lesion depth as it was described earlier, the patient’s risk profile, enamel surface integrity, and lesion activity are now the main focus of modern treatment strategies [28]. 

## 4. Initial Caries Detection Methods

As with any other disease, the detection of advanced disease signs is usually easier than the detection of the earliest signs. The importance of early detection of enamel lesions can be summarized in two points: 1: Increasing the chance of detecting non-cavitated lesions which allows more possibility for non. invasive and micro-invasive treatments to be effective. 2: The possibility of monitoring the lesion progression after providing non-invasive and micro-invasive measures [58,59,60,61].

The detection of carious lesions involves identifying the indications of dental caries. These lesions can be clinically detected at various stages, such as non-cavitated, micro-cavitated, and cavitated. Through a thorough clinical examination of a dry, clean enamel surface, it is possible to observe these lesions in occlusal fissures, on the vestibular surface, and even on proximal surfaces [13].

Complementary detection tools, including radiography, optical, and electrical methods, can also be employed to detect caries lesions. In vitro caries lesion detection methods encompass histology, polarized light microscopy, transmission, scanning electron microscopy (SEM), and confocal laser scanning microscopy (CLSM) [18].

Before the introduction of bitewing X-rays by H.R. Raper in 1925 [62], proximal caries lesions were primarily detected through clinical and tactile examinations, a method suitable for identifying cavitated caries. For much of the past century, the standard approach for caries detection and treatment planning in dental practice involved a combination of visual, tactile, and radiographic examinations [63,64].

Identifying enamel cavitation is crucial for the management and monitoring of the disease. While it is relatively straightforward for occlusal lesions, detecting cavitated lesions on proximal surfaces is more challenging. Clinical examination alone has been shown to identify only 12–50% of cavitated proximal lesions [31].

The use of a sharp dental probe during clinical examinations has been criticized for over a decade as an inappropriate tool for assessing dental lesions [65,66]. The sharp probe tip can cause irreversible damage to demineralized enamel and create cavities. Despite this, many general dentists still use such tools for tactile examinations [67]. The use of a ball-ended explorer has been recommended as a safer and improved method for caries assessment. Although using sharp probes can better distinguish between varying levels of roughness, the ball-ended probe is likely safer due to its lack of a sharp tip [68,69].

Visual examinations aimed at detecting non-cavitated lesions reportedly exhibit varying sensitivity (0.20–0.96), specificity (0.50–1.00), and diagnostic inconsistencies among examiners. The challenge of early detection of proximal lesions based solely on clinical examination led to a reliance on radiographic examination, primarily bitewing radiographs. However, radiographs exhibit low sensitivity (0.14–0.38) and high specificity (0.59–0.90) in detecting dentin caries lesions and are often considered inadequate for reliable early caries detection [54,56,70].

It has been suggested by Wenzel et al. that 30–40% of enamel must demineralize before an enamel lesion is visible on radiographs [64]. Yang and Dutra [71] have also indicated that as much as 40–60% of tooth decalcification is required for a lesion to be detectable on a radiograph. Furthermore, bitewing radiographs cannot determine the status of enamel surface integrity (cavitated or not) or the activity level of the lesion [54,64].

In recent decades, the overall improvements in dental hygiene and regular patient follow-ups led to important global changes in caries representation, with more concealed lesions and the rise of so-called ‘hidden caries’ [72]. Those changes have prompted the need to detect and stop occlusal and proximal carious lesions at an earlier stage [73,74]. 

A recent systematic review and meta-analysis concerning bitewing radiography for caries detection established the accuracy of radiographic caries detection for cavitated proximal lesions and also seems suitable to detect dentine caries lesions, yet new methods are needed for the detection of early enamel caries [54]. 

It can be concluded that while visual inspection and intraoral radiographs are fundamental in dentin caries detection, they seem to have suboptimal sensitivity for early caries lesions, hence the need for more sensitive tools [75].

### 4.1. Light-Based Caries Detection and Monitoring Methods

Numerous non-invasive methods for initial caries detection have been developed. These tools are mostly based on the mineral and optical properties of enamel and the difference between healthy and demineralized enamel [56].

These non-invasive early caries detection methods involve the use of different wavelengths and technologies to detect caries. A few examples include laser fluorescence devices, electrical caries monitoring, photo-thermal radiometry, fiber-optic transillumination, and near-infrared transillumination and reflectance [5,7,55,70,76,77,78]. 

#### 4.1.1. The Use of Fluorescence in Caries Detection and Monitoring

Fluorescence is one of several mechanisms through which materials can emit light upon suitable activation. In the realm of dentistry, light-induced fluorescence harnesses the innate fluorescence of teeth to distinguish between caries and healthy dental tissues [79].

Numerous imaging techniques developed for caries detection rely on the fluorescent response of organic components within teeth. These devices are categorized into red, blue, and green light-based systems [80]. The emitted fluorescence light’s color always differs from the excitation light due to variations in energy, wavelength, and photon energy. Consequently, violet or blue excitation light yields emissions in green, orange, or red, which are longer wavelengths of visible light. Similarly, visible red excitation results in emissions in the near-infrared region. This relationship is known as the Stokes’ shift [81,82].

Red fluorescence is employed in devices like DIAGNOdent and DIAGNOdent pen from KaVo in Biberach, Germany. These instruments employ a small laser with an excitation wavelength exceeding 655 nm. The device’s tip emits the excitation light and collects the resulting fluorescence, with results displayed on a continuous scale ranging from 1 to 99 [83,84]. These devices operate on the principle that carious tissue emits more fluorescence than healthy tissue due to the presence of bacterial by-products (porphyrins) [56].

Green fluorescence is utilized in devices that utilize quantitative light-induced fluorescence (QLF). QLF relies on the fluorescence characteristics at the green-yellow end of the spectrum (around 370 nm). This emitted fluorescence or refracted light is captured by the device, allowing measurements of tooth fluorescence (quantitative light-induced fluorescence) to be taken, often expressed as an average loss of fluorescence symbolizing lesion depth (commonly labeled as ΔF and assigned a value on a numeric scale) [79,85].

Blue fluorescence devices operate within the blue/violet end of the visible light spectrum (400 nm to 450 nm) and produce red luminescence in regions indicating bacterial activity, often associated with dental caries. Conversely, sound or healthy areas of the tooth continue to fluoresce green [86]. Various software-dependent devices in this category offer imaging of luminescence regions. Examples include DIAGNOcam vision full HD by Kavo in Germany, VistaProof by Durr Dental, and SoproLife by ACTEON in France. While some cameras employ software to generate a numeric score ranging from 0 to 5, many rely on the operator’s interpretation of imaging program findings, classifying them into groups ranging from sound to visible dentine caries [87,88].

This technology has demonstrated an estimated sensitivity of 0.70 (with a 95% confidence interval of 0.64 to 0.75) at a fixed median specificity of 0.78, along with an intraclass correlation coefficient (ICC) of 0.96 [80]. In addition, both intra- and inter-examiner agreements were found to be 0.93 and 0.92, respectively [56]. These results signify its potential to mitigate the risk of diagnostic discrepancies that often occur when assessing non-cavitated early caries lesions solely through visual and radiographic means [56].

At a wavelength of 405 nm, violet light emits strong signals from numerous bacterial species involved in the caries process. Notably, Lactobacilli, which are secondary colonizers of carious lesions, emit more visible red fluorescence than mutans streptococci. Furthermore, Actinomyces odontolyticus exhibits robust porphyrin fluorescence [89]. Protoporphyrin IX, a derivative of hemoglobin and a component of the heme biosynthetic pathway, is involved in this process. As these porphyrin derivatives are absent in healthy tooth structures, they serve as markers for bacteria associated with dental caries. The excitation of fluorescence in these fluorophores is most pronounced in the visible violet-blue range (390–420 nm), with the peak excitation occurring at approximately 405 nm [90,91,92].

Red light emissions under violet light excitation are well-suited for detecting key cariogenic bacteria (102). However, it is essential to consider that the fluorescence characteristics of specific bacterial species may vary based on the nutrients present in their environment, such as blood and associated metalloporphyrins [93,94].

These discoveries have led to the preference for 405 nm violet light-emitting diodes (LEDs) as the illumination source of choice. They have been integrated into various complementary devices for caries detection, including intraoral cameras, pen-shaped illuminators, microscopes, and dental high-speed handpieces. These tools assist in caries detection during clinical examinations and even during the excavation of carious lesions [95].

These tools can provide information about lesion size, fluorescence loss, bacterial activity by the presence of red fluorescence, and staining intensity. These devices could also be used to detect and assess mature deposits of dental plaque biofilm more than 24 h old, as these have high levels of porphyrins and give strong red fluorescence when excited by violet light [95]. 

**Figure 2:**
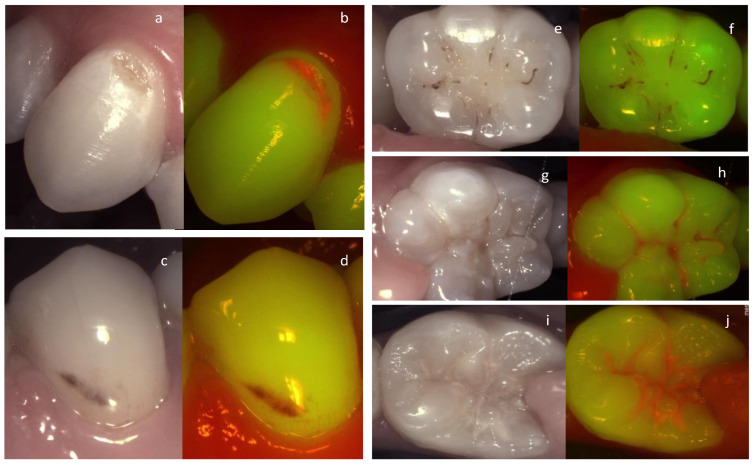
An active cavitated vestibular lesion on the upper canine (**a**,**b**) can be easily identified on the fluorescence image due to the bright red fluorescence. On the lower premolar (**c**,**d**) the lesion is considered arrested clinically according to the Nyvad criteria. The lack of red fluorescence, the brown area of fluorescence loss, and the staining confirm this on the fluorescence image. The same trend can be observed in the images of the molars on the right. Arrested occlusal lesion (**e**,**f**) active occlusal lesions (**g**–**j**).

It can be concluded that besides caries detection, fluorescence can be used to evaluate and monitor lesion activity which has been demonstrated in multiple studies [96] (Figure 2). 

Moreover, the visual aspect of plaque fluorescence on the documented images can be of great advantage in patient management and motivational discussion. The images are more comprehensible for the patients and allow a higher level of engagement when explaining the need for better management of the biofilm. 

It must be noted that there are some drawbacks to light-induced fluorescence for caries detection when used for the sole objective of caries detection. The carious lesions can be confused by the presence of contamination such as blood, calculus, or plaque which might cause a false positive reading leading to an over treatment in unexperienced hands. [79] 

#### 4.1.2. The Use of Near-Infrared Transillumination in Caries Detection and Monitoring

One of the oldest alternative caries detection methods after radiographs is the use of transillumination [97,98]. The concept relies on the enamel’s optical properties that are modified by the slightest change in enamel porosity, which translates into increased scattering when light passes through the enamel [13,55,97].

During light scattering, the direction of a photon changes without loss of energy. The incident light deviates from its path after it interacts with obstacles in the tissue through which the light passes, i.e., enamel demineralization in this context [99]. Scattering is highly wavelength sensitive, shorter wavelengths scatter more than longer ones [100,101]. Consequently, caries detection methods employing wavelengths in the visible range (400 nm to 700 nm) are highly limited by scattering [102,103].

Fiber optic transillumination (FOTI) was first presented for the detection of proximal caries in the early 1970s [104,105,106,107], the technology is sensitive to early changes in the enamel structures and it was described as a useful aide to be combined with radiography and clinical examination [108,109]. However, it has been determined that FOTI diagnosis by the naked eye can be subject to great inter- and intra-examiner variation [110]. This led to the development of digital fiber optic transillumination imaging (DIFOTI) in the 1990s [111,112] to enable image capturing and enhance the possibility of lesion monitoring over time. This technology was investigated and validated in multiple in vitro and in vivo studies. Compared to other detection tools, it showed a high sensitivity for early enamel lesions detection and the potential for monitoring such lesions over time [31,111,113,114,115]. The technique has been comprehensively described and compared with radiography and clinical tactile evaluation with some contradictive results [7,114,116].

The development of near-infrared transillumination for caries detection started around 1995 [103]. This method uses near-infrared wavelength interactions with the dental substance allowing visual discrimination between healthy and demineralized tissue [103,117,118,119]. Fried et al. demonstrated that dental enamel becomes highly transparent when illuminated with NIR light, while dentine scatters strongly the visible and NIR light. This implies that the technique is appropriate to study caries lesions in enamel and less so in dentine [103]. 

Imaging with NIR light at 1310 nm has demonstrated considerable potential for the detection of early demineralization compared to dental X-rays [119,120]. The technique was mainly described for proximal caries but it can additionally detect occlusal caries and cracks [121]. Several studies have shown NIR to have higher sensitivity than BW to detect both proximal and occlusal caries and may be used for monitoring [122,123,124,125,126]. Over the past ten years, NIRT imaging was further examined for multiple indications including primary and secondary caries detection on occlusal and proximal surfaces, early caries monitoring, caries removal, caries detection and monitoring under sealants, and guided caries removal [55,117,118,127,128]. 

Compared to the BW radiographs, the occlusal viewing angle of the images makes it easy to localize the exact position of proximal decay in the bucco-oral dimension to decide on the most straightforward access to the carious lesion from the occlusal surface, reducing unnecessary removal of healthy tissue.

In 2012, the DIAGNOcam device (KaVo, Biberach, Germany) using 780 nm near-infrared transillumination technology became available, followed by the new version (DIAGNOcam Vision full HD) combined near-infrared transillumination with clinical images and fluorescence in 2021. The combination of the three images provides an undisputable advantage for the clinician as the combination of the information obtained from the three images at the same time enhances the chances of making an accurate diagnosis and hence an appropriate management plan. Over the last two years, new tools such as 3D oral scanners have integrated these technologies as well [129,130,131].

For transillumination, the camera is equipped with two flexible silicon extensions that illuminate the tooth from both the vestibular and oral directions. Near-infrared (NIR) light is employed, passing through the periodontal and dental tissues, with an infrared-sensitive camera capturing images from the occlusal surface of the examined tooth. This near-infrared wavelength at 780 nm falls within the optical window of tissues. Utilizing a wavelength within this optical window ensures superior light transmission through tissues, allowing for deeper penetration and enhanced image quality compared to visible light [121].

**Figure 3:**
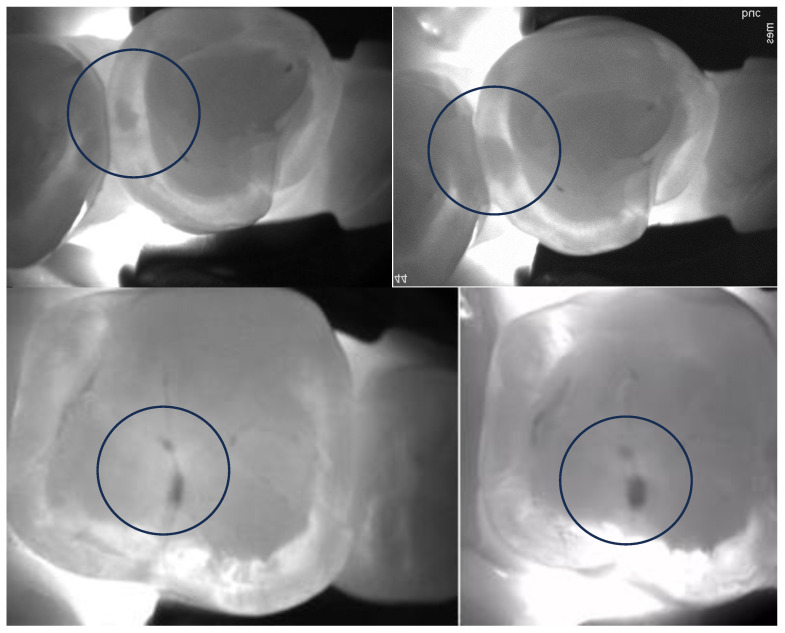
Encircled occlusal (**upper** images) and proximal (**bottom** images) lesion progression can be confirmed after approximately 2 years of monitoring.

Recent reports and reviews on near-infrared transillumination technology suggest that it may emerge as a valuable alternative to bite-wing radiography for the early detection of proximal caries, especially during enamel lesion monitoring in recall examinations (see Figure 3). Notably, it does not involve ionizing radiation, so there are no restrictions on its frequency of use. Supporting this concept, a study revealed that NIRT imaging’s diagnostic outcomes are on par with bitewing radiographs [57,132]. It was demonstrated that lesions detected in NIRT images closely correlate with X-ray images and clinical assessments (NIRT vs. X-ray image 97%; NIRT vs. clinical 96%) [57,132,133]. 

Furthermore, a recent clinical study found that NIRT devices, when compared to BW radiographs, are more adept at detecting early proximal enamel lesions [134,135]. Additionally, when the NIRT device was used to detect proximal caries with two-year intervals between readings, the reproducibility of caries detection was excellent compared to BWs of the same patients. NIRT appeared to identify more enamel caries lesions than radiographs, revealing approximately four times as many lesions reaching the enamel–dentin junction in comparison to BW radiography [60]. Similar findings have been reported recently [134,136], highlighting the high reliability of NIRT and its strong agreement with clinical and radiographic examinations in detecting dentin caries lesions.

In conclusion, caries detection using NIRT could be seamlessly integrated into daily clinical practice as a primary method for early caries detection, complementing clinical examinations. Subsequently, BW radiographs may be obtained to determine the depth of carious lesions once dentin lesions have been identified through NIRT images.

**Figure 4:**
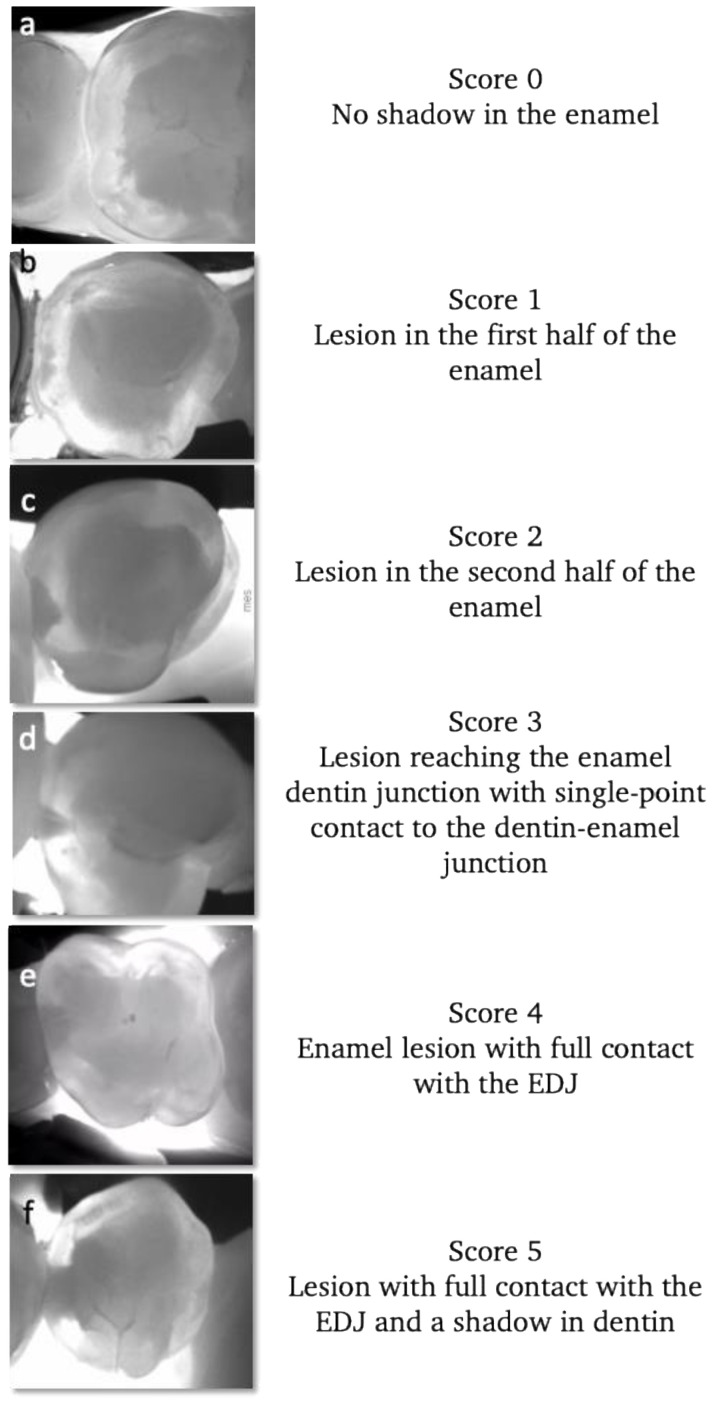
Examples of different stages of proximal lesions detected using near-infrared transillumination. Starting with healthy enamel (**a**), enamel lesions (**b**–**d**) and dentin lesions (**e**,**f**). The progression of proximal lesions can be monitored and assessed during recall based on this scoring.

Near-infrared transillumination empowers clinicians to detect proximal lesions that might escape clinical observation and distinguish between lesions confined to the enamel and those that have progressed to the enamel–dentin junction (as depicted in Figure 4). Consistent monitoring using this technology can aid clinicians in tracking lesion activity and the pace of their progression. This, in turn, allows for the customization of preventive measures and the early adoption of non-invasive caries management on an individual basis [135].

Again, it is important to stress that the heightened sensitivity of these new technologies for early caries [113,137] may carry the risk of overtreatment, especially if treatment decisions are rooted in outdated principles. If dentists adhere to old treatment approaches designed for radiography, where operative intervention is indicated once the lesion reaches the enamel–dentin junction, overtreatment becomes unavoidable [138,139,140]. 

It is essential to realize that these above-mentioned technologies, like any others, have their disadvantages or shortcomings as well. None of them can be recommended as a single diagnostic tool for caries detection.

**Figure 5:**
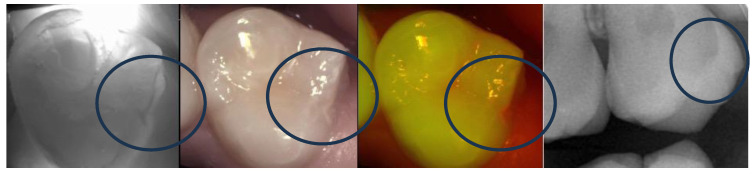
Occlusal lesion of the occlusal distal fissure (encirceled) is not very clear on the NIR image while the X-ray shows clear dentin extension. However, the fluorescence image indicates high activity of the lesion.

For NIR, experience shows that some proximal caries can be missed depending on the shape of the proximal surface and the location of the lesion. The closer the lesion is to the cervical area the less chance to detect it. Some teeth, like the first lower primary molars, are very difficult to image [141] and proximal caries can be missed. It was also noticed that some occlusal caries reaching the dentin could be missed depending on where the lesion formed and the angulation of the tooth (Figure 5) [142]. 

### 4.2. New Developments for Lesion Assessment

To assess lesion activity, few available techniques besides systems combining visual and tactile criteria are available. Here, we discuss devices based on thermal imaging, dye-enhanced fluorescence, and bioluminescence. 

#### 4.2.1. Thermal Imaging and Optical Coherence Tomography for Lesion Activity Assessment and Monitoring

Researchers have devoted significant time to the assessment of lesion activity using novel tools. Historically, histological sections, transverse micro-radiography (TMR), and polarized light microscopy (PLM) have been employed to gauge lesion activity by visualizing the highly mineralized surface layer. However, these methods entail sample destruction and are unsuitable for clinical applications [143].

It has been demonstrated that when the mineral content of the outer surface layer is sufficiently high, it becomes more transparent, allowing for its visualization and measurement through optical coherence tomography (OCT). Several studies, both in vitro and in vivo, have substantiated OCT’s ability to detect this transparent surface zone [144,145]. Additionally, systems have been devised to automatically identify the transparent surface layer of enamel lesions after exposure to remineralization solutions and measure its thickness [146].

Recent developments in caries activity assessment have explored changes in thermal imaging during the dehydration of the lesion in question. Typically, caries lesion arrest is accompanied by remineralization in the outer surface layers, which are highly mineralized. This layer impedes the diffusion of fluids between the surface and the bulk of the lesion. It is presumed that the rate of water diffusion out of the lesion reflects the degree of lesion activity. In other words, the loss of water from the porous lesion can alter the reflectivity and light scattering from the lesion. Studies have demonstrated that optical changes due to the loss of water from porous lesions can be employed to assess lesion activity using fluorescence and short-wavelength infrared (SWIR) imaging [147,148,149,150]. Several studies have shown that thermal imaging can detect alterations in tooth mineralization. Researchers like Kaneko et al. and Zakian et al. established the differentiation of lesions on coronal surfaces from sound enamel in thermal images [151,152]. Subsequent in vitro studies revealed that temperature changes resulting from water loss in porous lesions can also be used to gauge lesion activity through thermal imaging [148,149,150]. Notably, thermal imaging during lesion dehydration proved more effective than near-IR imaging for evaluating lesion activity on root and dentin surfaces in previous studies [149]. A recent clinical study has indicated that thermal imaging can be used to assess the activity of root caries lesions [153].

The surface zone that forms during remineralization in an arrested caries lesion is directly related to its permeability and activity properties. When correlated with results from short-wavelength infrared (SWIR) imaging during dehydration measurements and OCT measurements, it was observed that even minor changes in surface layer thickness could lead to significant alterations in fluid permeability in remineralized lesions [149].

These new tools can be of interest to monitor the changes in lesion activity over time after the application of preventive and lesion control products such as silver diamine fluoride or fluoride varnish [154,155].

#### 4.2.2. Dye-Enhanced Laser Fluorescence

The use of a fluorescent dye for the detection and monitoring of early caries lesions has been described in the late 1990s. This technique is based on the dye’s ability to penetrate the porous structure of such lesions through capillary action. A method known as Dye-enhanced Quantitative Light-induced Fluorescence (DEQLF) is employed for imaging and quantifying fluorescence by applying a fluorescent dye onto caries lesions and imaging them. The dye of sodium fluorescein has been used in ophthalmology for a long time, it is also used in dentistry as a plaque-disclosing agent [156].

In active lesions, penetration of the fluorescent dye into the lesion results in increased intensity of the autofluorescence.

DEQLF stands out as a promising method for objectively assessing lesion activity. Its potential extends to becoming a valuable tool for both single-observation assessments and longitudinal monitoring of lesion activity in clinical practice. Furthermore, the simplicity of the DEQLF procedure makes it accessible, enabling even dentists with limited experience in evaluating early caries lesion activity to use it effectively in clinical settings and for larger cohorts.

#### 4.2.3. Bioluminescent Photoproteins to Detect Lesion Activity in Enamel

In 2005, Professor Pitts and Dr. Longbottom identified the unmet need to assess the activity of non-cavitated carious lesions in a single dental examination. Inspired by the discovery of bioluminescent photoproteins reacting to free calcium ions (Ca^2+^), they explored the potential of using such photoproteins to detect Ca^2+^ released during the demineralization process of caries development [157,158]. Photoproteins, naturally occurring bioluminescent proteins emitting light, were sourced from marine organisms like jellyfish. Unlike fluorescent markers, photoproteins do not require an excitation source. The vision was to create a device aiding dentists in managing caries by identifying active lesions in a single visit, leading to targeted preventive care. In collaboration with ‘Lux Innovate Ltd.’, a biotechnology company began to develop the luminescence system and photoprotein. Patent applications were initiated in 2007 and 2011.

The recent publication of clinical study results, involving 110 participants aged 7–74 years, reveals significant findings. The study demonstrates that the Calcivis Imaging System exhibits a high level of agreement in distinguishing tooth surfaces clinically identified as having active enamel lesions (ICDAS code 2/3) from sound sites, which are biochemically equivalent to inactive lesions. Moreover, the study affirms the system’s safety for clinical use. In this in vivo investigation, among the 90 teeth assessed as sound or equivalent to inactive lesions, 88 exhibited no bright light (bioluminescent signal), indicating a negative percentage agreement of 97.8%. Conversely, of the 86 teeth identified as having active lesions, 78 displayed a bright light (bioluminescent signal), resulting in a positive percentage agreement of 90.7% [159].

The Calcivis System offers another advantage over indicators focusing on bacterial by-products or components. In addition to its application in detecting carious lesions, the system can identify enamel demineralization caused by erosive tooth wear—a distinct condition involving non-carious demineralization. This advanced capability enables dentists to enhance the implementation of risk-adjusted preventive clinical management for both caries and erosive tooth wear, thereby providing a more comprehensive approach to patient care [157].

#### 4.2.4. Fluorescent Starch Nanoparticles

The use of nanoparticles for medical applications has acquired considerable interest, particularly for applications in drug delivery, diagnostics, and imaging. Recent studies have shown that fluorescent cationic starch nanoparticles (FCSNs) are able to successfully penetrate the subsurface of artificially prepared carious (active) lesions [160]. the FCSN technology was developed to target active lesions by virtue of their open surface porosity, but not inactive lesions which have sealed enamel surfaces.

Intended for application as a mouth rinse during a routine examination post-dental cleaning, FCSNs are subjected to illumination using a standard blue light curing lamp with an orange filter—readily accessible in dental offices. This process yields a vivid fluorescent yellow-green visualization of early enamel lesions, enhancing the dentist’s visual examination capabilities [160].

GreenMark Biomedical Inc. has introduced LumiCare™ Caries Detection Rinse (LC Rinse, MI, USA), a diagnostic oral rinse incorporating proprietary fluorescent starch nanoparticles. This oral rinse facilitates the illumination of active initial caries lesions, indicated by a positive LC Rinse response when exposed to a dental curing lamp [160].

In experiments involving extracted teeth, LC Rinse exhibited high reproducibility and accuracy in detecting occlusal caries, demonstrating notable sensitivity and specificity when compared to histology [161].

A clinical study showed that LC Rinse can distinguish between active caries, inactive caries, and hypomineralization, and can augment caries detection with high sensitivity, specificity, and diagnostic accuracy [162].

Recent research has identified LC Rinse’s utility in conjunction with Artificial Intelligence (AI)/Computer Vision as an objective tool for caries diagnosis [161,163].

LC Rinse currently holds FDA clearance for aiding dental professionals in visualizing caries lesions.

### 4.3. Methods to Differentiate between Cavitated and Non-Cavitated Lesions

The gold standard in clinical research for detecting cavitated proximal lesions is the use of orthodontic elastics for temporary separation. This technique provides visual access for the inspection of the proximal surface and may be combined with taking an impression of the surface [164]. This technique, however, requires two visits and may cause some discomfort during the first few hours, these disadvantages make it unpopular among dentists and patients.

In a pilot investigation, a radio-opaque paste was suggested for placement between teeth to reveal cavities on proximal surfaces when identified through bitewing radiographs [165]. Furthermore, a recent study explored the potential use of a near-infrared absorbent dye, such as Indocyanine green, as a contrast agent for discerning cavities in proximal lesions when using near-infrared transillumination images. The results were promising in distinguishing between cavitated and non-cavitated natural proximal lesions in vitro [166]. Another recent study examined the feasibility of employing near-infrared (NIR) reflectance and transillumination images for the purpose of cavity detection [167].

Nevertheless, the above-discussed ideas are still in the preliminary stages. Without another possibility for differentiating between cavitated and non-cavitated proximal lesions, even modern caries management approaches base the decision-making strategy on clinical examination and lesions’ radiographic depth [28].

Adding to all these tools introduced above, one of the most recent developments is the introduction of artificial intelligence (AI) applications in medical imagery analysis. This technology could help harmonize and standardize the detection of caries on radiographs and different imaging technologies like near-infrared transillumination providing a new promising research path [168,169].

It must be stated that in low-risk populations, with low prevalence and slow-progressing lesions, the risk of over-detection is likely to occur. There are few lesions to detect [73,170], and even if lesions are identified, they are mostly inactive and do not require active therapy [171]. This is why it is important to adopt the most recent guidelines and to adapt the diagnostic strategy (diagnostic tool and the interval to apply it) to an individual’s personal caries risk. These tools will be most useful in high-risk individuals if needed in shorter intervals to monitor lesion activity and reduce the need for frequent irradiation [172].

## 5. A Proposal of a Workflow Integrating near Infrared Transillumination and Fluorescence into the Caries Management System

Nowadays, dentists have more treatment options and evidence available to support them than ever before.

We finally seem to be moving toward more minimal-intervention, evidence-supported, and personalized treatment options that focus on promoting and maintaining oral health.

Considering all the aspects discussed earlier, we can see that we have all the elements required for a comprehensive management of the early carious lesion. However, to our knowledge, no management system described in the literature has actively integrated near-infrared transillumination and fluorescence into the workflow of the oral care plan and therapeutical decision making. Integrating these tools can allow the early detection of lesions that are clinically undetectable (sub-clinical), can provide an indication of site activity, may significantly increase the effectiveness of available management systems, and help target at-risk patients early before the symptoms are detectable by the naked eye or on radiographs.

As an example and based on the available evidence in the literature and the clinical experience gained over the last decade, this paper proposes a comprehensive management approach for occlusal caries combining clinical scoring based on simplified ICDAS, NIRT image scoring, activity assessment based on clinical criteria and fluorescence imaging signal, and caries risk assessment using tools like Cariogram or CAMBRA.

This approach can help dentists provide personalized care plans by choosing elements from evidence-based management options (noninvasive, micro-invasive, and restorative) and can guide reevaluation (active monitoring) and follow-up possibilities.

The scoring systems proposed are based on published scoring models for clinical [20], NIRT [61,173], and fluorescence [174] with some modifications and adaptations to improve the workflow.

### 5.1. Clinical Scoring

Based on the ICDAS system and the ICCMS [20], the scoring should be performed under sufficient light and with appropriate magnification. Patients should ideally brush before. Carious lesions are divided into the following:

(a) Early lesions, which include code 1 and 2 ICDAS: enamel lesions.
(b) Moderate lesions, codes 3 and 4 ICDAS: micro cavitated enamel lesion or non-cavitated dentin lesion.
(c) Advanced or extensive lesions, codes 5 and 6 ICDAS: lesions presenting larger cavitations with visible dentin.

Radiographically, early and moderate lesions (codes 1–4 ICDAS) are often not visible on BW X-rays [61] or if visible they are limited to the first third of dentin, while advanced lesions extend into the inner third and further [175].

The dilemma of professional cleaning before or after the examination is still not clear to most practitioners. To do a proper scoring based on clinical examination, the tooth needs to be clean and dry. Depending on the patient’s hygiene, lesions can be covered with plaque or calcified organic matter which will mask the real extent of the lesion and impact the validity of the clinical examination scoring. However, professional cleaning before the examination will eliminate all plaque; hence, the evaluation of lesion activity based on the presence of plaque cannot be achieved and the appreciation of patients’ capacity to eliminate plaque is not evaluated.

If the patient is asked to brush before the examination, the plaque remaining will indicate their actual capacity to clean. And the remaining plaque could be removed by the practitioner when examining certain teeth.

The fluorescence and near-infrared transillumination images with additional information confirming the clinical image or even revealing lesions not visible clinically can be extremely valuable for early and moderate lesions.

### 5.2. Near-Infrared Transillumination Scoring

**Figure 6:**
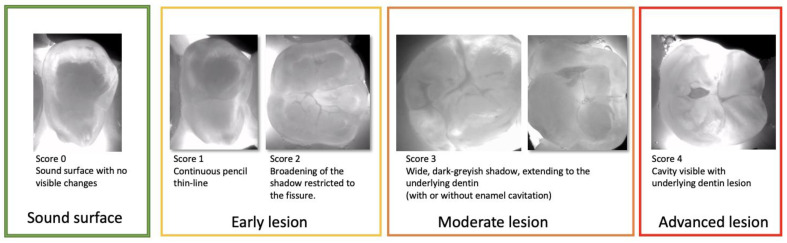
Near-infrared transillumination scoring. Occlusal lesion progression on NIR images is observed by the appearance of the dark lines within the fissures in the early lesions, the widening of the fissures in the moderate lesions reaching the first third of dentin.For sever lesions with dentin cavitation the extension into the dentin cannot be estimated.

Early signs of demineralization are easily visible on NIRT images (Figure 6). Very early (sub-clinical) enamel lesions are seen as very thin dark lines that follow the occlusal fissure system, while darker and wider lines represent lesions advanced into the enamel dentin junction. For moderate lesions, a wider shadow under the enamel indicates the involvement of the dentin below. These cases can have enamel cavitation corresponding to a score of 3 ICDAS or without cavitation corresponding to a score of 4 ICDAS.

The lesion contrast in transillumination increases with lesion depth. The contrast of the severe lesions is significantly higher than the shallower lesions [176]. A drawback of the near-infrared transillumination method is the lack of information concerning the depth of the lesion when it reaches the dentin. Bitewing X-rays may be justified in these cases to assess the lesion depth and to monitor it.

This NIRT scoring at this level of early lesions may be considered an over-detection. When detected on the NIRT image, scores 1 and 2 may be clinically scored as code 0,1 or 2 ICDAS.

These lesions should alarm the dentist that this surface is at risk. The therapeutic impact we hope for here is increased prevention and prophylaxis to arrest the lesion. Longitudinal monitoring will allow activity assessment. If the lesions progress into score 3 despite the non-invasive management, a micro-invasive approach may be considered.

### 5.3. Fluorescence Image Scoring

While using ICDAS and Nyvad criteria [40] is useful to detect and assess the activity of early occlusal lesions, a surface should be thoroughly cleaned and dried for 5 s. This requirement is not always applied by practitioners and caries screening on a population level.

However, when the visual examination is combined with fluorescence images, it can be easier to detect and monitor early lesions. It must be understood that without cleaning the surface the fluorescence image must not be used to detect caries; the presence of plaque or coloration may falsely be interpreted as caries. However, if we consider the presence of cariogenic plaque (red fluorescence signal) on the occlusal surface an indication of lesion activity as described by Nyvad, this method can be very helpful in assisting the assessment and monitoring of lesion activity.

**Figure 7:**
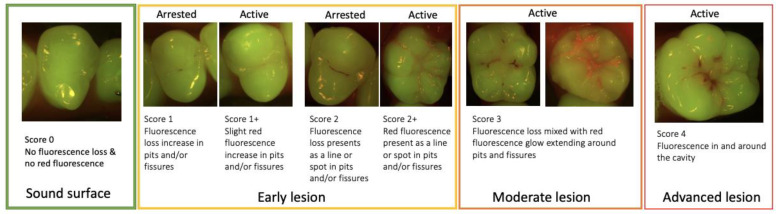
Fluorescence image scoring.

In the scoring system described below (Figure 7), we assess the lesion activity at each stage to help guide the preventive care plan. Moderate lesions are considered active (often mixed) [177] and the management of these lesions will mostly depend on the patient’s risk assessment.

**Figure 8:**
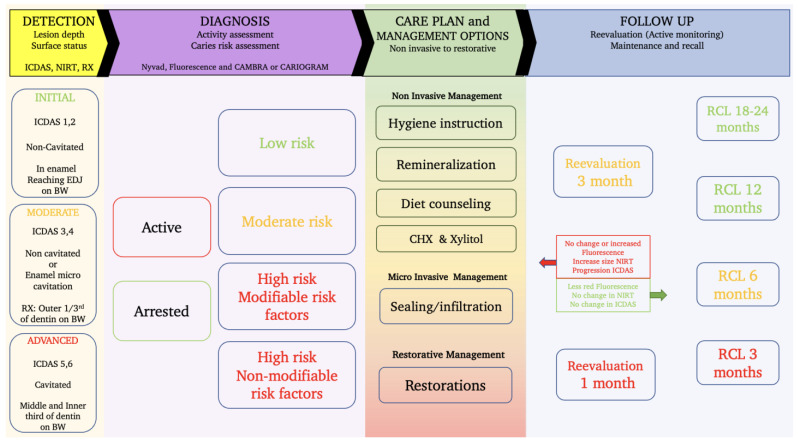
A workflow for caries management and care plan development integrating the use of NIR and fluorescence to assist the detection and diagnosis phase as well as the revaluation and monitoring aspect.

The figure below (Figure 8) provides an overview of the required steps from the detection, the diagnosis, and the management options to the reevaluation and recall possibilities.

Figure 8 is mainly based on the latest guidelines for caries management; it provides a full panel of the required steps and options to help guide the dentist through the process [178].

The detection of the lesion is carried out based on the ICDAS system combined with near-infrared transillumination to detect the subclinical hard-to-see early lesions, and the use of X-rays can complete the examination for determining dentin lesions depth.

Lesion activity assessment is achieved based on the fluorescence images combined with the Nyvad criteria. Even though the red fluorescence signal may not be from the lesion directly and from the plaque covering it, the presence of cariogenic plaque revealed by the red fluorescence indicates that the lesion is active. We can consider that the presence of red fluorescence on a sound surface or an early enamel lesion indicates that the surface is at risk of lesion development and progression. Cleaning the surface mechanically using a rotative brush or air abrasion (airflow) will reveal the actual state of the tissue underneath. The impact of a patient adhering to a better brushing technique for example can be monitored in this way. Arrested lesions appear as a darker line or area as a result of fluorescence loss due to enamel demineralization (Figure 2).

Depending on the patient’s caries risk profile obtained using CARIOGRAM, CAMBRA, or other caries risk assessment tools, a proposition of a care plan is put together by choosing the suitable management options.

In the majority of situations, it is recommended to start the care plan with non-invasive preventive measures like reviewing the brushing technique and maybe introducing interdental cleaning methods and simple self-care instructions. For patients with higher caries risk, we can consider increasing the remineralization chances by introducing brushing two to three times per day with 1.1% (5000 ppm) NAF toothpaste, adding Xylitol chewable tablets (6 g/day), encouraging the home application of fluoride gel or frequent in-office varnish application (5% NAF), or the use of calcium–phosphate-based paste (MI Plus” GC or Tooth Mousse”, GC Tokyo, Japan), or introducing chlorhexidine mouthwash: 0.12%: 1 min every evening for 1 week per month. For patients identified with a high quantity or frequency of sugar consumption, it is essential to take the time and provide some diet recommendations.

The goal is to help arrest the lesions detected, and to modify the patient’s risk category by working on the main risk factors identified (bad hygiene, lack of remineralization agents, diet, or others).

Active monitoring by frequent reevaluations using the same tools including NIRT images, fluorescence images, Nyvad criteria, ICDAS, and CRA is necessary to establish if the preventive noninvasive measures are achieving the desired goal.

A positive outcome is confirmed by reducing the red fluorescence signal on the fluorescence images, changes in the clinical aspect of the lesion (color, texture, less plaque clinically, less bleeding on probing near the lesion), and changes in the risk profile observed through the CARIOGRAM risk factors distribution.

In case no changes are observed after the reevaluation period using the non-invasive measures, micro-invasive procedures like sealing and infiltration can be justified to arrest (or slow down) and isolate non-cavitated or even micro-cavitated lesions. For cavitated lesions, micro-invasive or classic restorations can be proposed.

In some cases, like pediatric patients with little cooperation or old patients with no aesthetic expectations, non-invasive alternatives like using silver diamine fluoride (SDF) or the hall technique should be considered when preventive measures are not working as expected.

A different approach should be considered for patients identified as high risk with non-modifiable risk factors such as patients suffering from xerostomia after irradiation, patients with low saliva flow due to medication or a chronic disease, patients with physical or mental handicaps limiting self-administrated oral care, and patients with known low compliance to regular dental visits due to socio-economical situation. For these patients, it is recommended to provide protection using sealing, infiltration, and micro-invasive restorations early upon lesion detection as the non-invasive measures might be less effective. This should be combined with an intensive recall and monitoring care plan.

Adapting the follow-up (Recall) interval is an essential aspect of the caries management plan. Closer intervals are required for moderate and high-risk patients to allow monitoring of the lesion activity and lesion progression and to help provide the patient with the support required until we can observe the change in behavior where the patient demonstrates that s/he adheres to the new routine and the signs of lesion arrestation are evident.

The new tools using near-infrared transillumination and fluorescence can make the reevaluation and follow-up appointments more informative for the patient and for the dentist. The images obtained allow us to monitor the early lesion activity and progression without the need for irradiation.

At the 3-month reevaluation, if no changes are observed in the plaque activity or an increase in the size of the lesion on the NIRT image, a sealing or infiltration technique is proposed to protect the lesion from further progression. In case the non-invasive measures worked and we can clearly see less plaque and no changes in NIRT images, we can propose a 6-month recall and adapt further follow-up appointments according to the findings.

**Figure 9:**
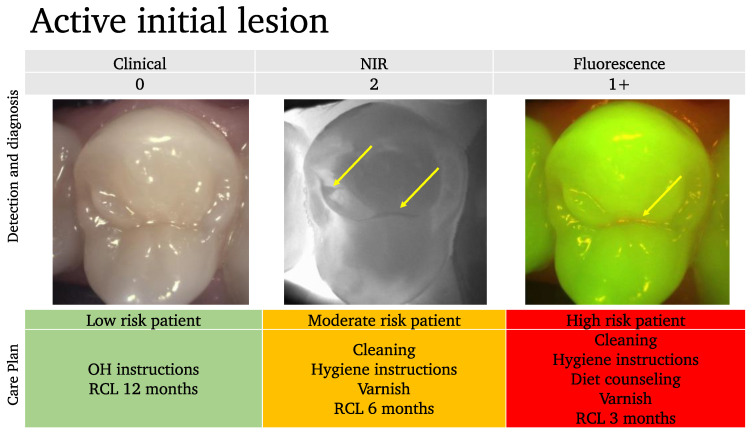
An example of an active initial lesion. While the clinical image suggests a healthy tooth, the near-infrared NIR and the fluorescence images show initial lesions (arrows). Depending on the patient’s risk assessment results, the care plan should be adapted.

To further illustrate the advantages of this approach, a few examples are considered below:

Example 1:

In this example (Figure 9), even though the clinical examination shows no lesion, we can clearly see the demineralized fissure on the NIRT image, in addition to the visible red signal on top of the demineralized area.

In this case, we can estimate that there is an early active lesion or at least a site that is at risk of further lesion progression.

Based on the risk assessment profile of the patient, the care plan is tailored, and the recall (RCL) intervals are adapted accordingly.

Without the NIR and the fluorescence scoring, this tooth would have been considered healthy and no further consideration for prevention would have been undertaken.

**Figure 10:**
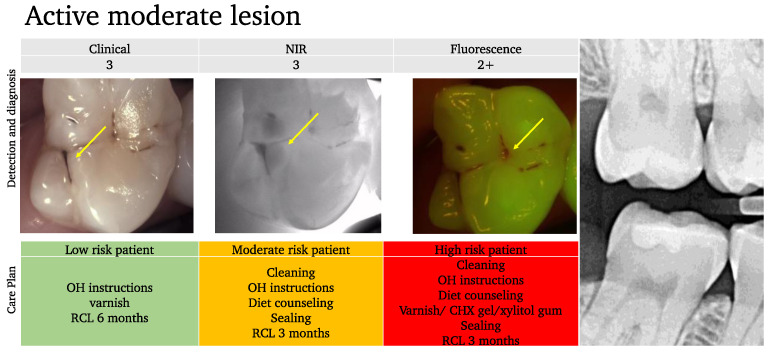
An example of an active moderate lesion. While the clinical aspect suggests an arrested lesion (arrow) and the bitewing X-ray shows no signs of an occlusal lesion on tooth 17, the NIRT image shows an extension into the dentin (arrow) and some fluorescence activity is also detected (arrow). Depending on the patient’s risk assessment results, the care plan should be adapted.

Example 2:

In this example (Figure 10), while the occlusal lesion seems to be arrested clinically, the NIR image shows an important extension into dentin with a mixed activity signal on the fluorescence image (dark spots with red signal around the lesion area).

With the classic concept, the lesion is considered arrested and since it is not visible on the BW radiograph, the management would have been to simply monitor for further progression. However, considering the extension into the dentin and the activity, we can provide a more elaborate care plan based on the risk profile and may consider sealing the lesion for moderate or high activity profiles.

**Figure 11:**
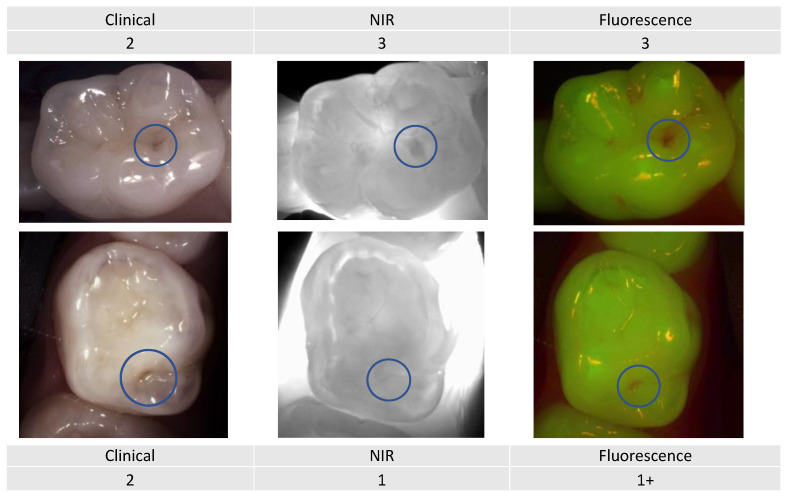
Two primary premolars presenting occlusal lesions (encircled) with similar scores clinically. The NIR and fluorescence images provide further information that will help guide the dentist to provide an adapted care plan depending on the caries risk profile.

Example 3:

Even though these two primary molars (Figure 11) would be clinically considered as an ICDAS score 2 (distinct change in enamel), the NIR image showing the extension into the dentin and the fluorescence activity signal reflects a very different image.

Combining the three detection and assessment methods with the risk assessment for decision making clearly changes the management approach depending on the patient’s caries risk assessment.

This approach, using all the described evidence on caries detection, diagnosis, and management combined with modern tools, aims to provide a patient-centered personalized care plan.

The concept requires the dentist and the patient to be invested in the process, and both need to realize that it can be a time-consuming and long-term collaboration which is clearly different from classic dentistry based on a drill-and-fill approach whenever a carious lesion is detected.

As with any management guideline, this approach requires validation through long-term clinical trials to assess the impact on patients’ oral health, the possibility of integrating the process into the dentist’s established workflow, as well as the financial impact on the dentist and the patient, and the cost-effectiveness of the concept.

## 6. Conclusions

The integration of advanced technologies such as near-infrared transillumination (NIRT), fluorescence-based imaging, and other modalities holds promising potential for optimizing patient-centered oral health care.

The substantial body of scientific evidence available supports the management of caries as a disease guided by the application of evidence-based principles and guidelines in an individualized and personalized manner.

It is imperative for practitioners on a global scale to assimilate this knowledge into routine clinical practice.
